# Facial Indicators of Positive Emotions in Rats

**DOI:** 10.1371/journal.pone.0166446

**Published:** 2016-11-30

**Authors:** Kathryn Finlayson, Jessica Frances Lampe, Sara Hintze, Hanno Würbel, Luca Melotti

**Affiliations:** University of Bern, Division of Animal Welfare, Bern, Switzerland; Eidgenossische Technische Hochschule Zurich, SWITZERLAND

## Abstract

Until recently, research in animal welfare science has mainly focused on negative experiences like pain and suffering, often neglecting the importance of assessing and promoting positive experiences. In rodents, specific facial expressions have been found to occur in situations thought to induce negatively valenced emotional states (e.g., pain, aggression and fear), but none have yet been identified for positive states. Thus, this study aimed to investigate if facial expressions indicative of positive emotional state are exhibited in rats. Adolescent male Lister Hooded rats (*Rattus norvegicus*, N = 15) were individually subjected to a Positive and a mildly aversive Contrast Treatment over two consecutive days in order to induce contrasting emotional states and to detect differences in facial expression. The Positive Treatment consisted of playful manual tickling administered by the experimenter, while the Contrast Treatment consisted of exposure to a novel test room with intermittent bursts of white noise. The number of positive ultrasonic vocalisations was greater in the Positive Treatment compared to the Contrast Treatment, indicating the experience of differentially valenced states in the two treatments. The main findings were that Ear Colour became significantly pinker and Ear Angle was wider (ears more relaxed) in the Positive Treatment compared to the Contrast Treatment. All other quantitative and qualitative measures of facial expression, which included Eyeball height to width Ratio, Eyebrow height to width Ratio, Eyebrow Angle, visibility of the Nictitating Membrane, and the established Rat Grimace Scale, did not show differences between treatments. This study contributes to the exploration of positive emotional states, and thus good welfare, in rats as it identified the first facial indicators of positive emotions following a positive heterospecific play treatment. Furthermore, it provides improvements to the photography technique and image analysis for the detection of fine differences in facial expression, and also adds to the refinement of the tickling procedure.

## Introduction

There is growing evidence that animals are capable of experiencing and expressing different emotions or affective states, including those of positive valence, such as happiness or pleasure [1,2]. Positive emotions have been identified as a critical marker for good animal welfare [1], thus investigating methods for recognizing both negative and positive emotional states in animals is essential. In animals, emotional states may be inferred from behaviour [3] including vocalisations [4,5], physiology [6], neurophysiology [7] or cognitive biases [8,9]. Moreover, different emotional states can be expressed through changes in facial expression, as previously shown in human and non-human primate studies [10–12]. Facial expressions have been recognized in a wide variety of animal species with dissimilar facial musculature, including dogs [13], horses [14,15], rabbits [16], mice [17] and rats [18,19].

In rats, the development of the Rat Grimace Scale has allowed to identify differences in facial expression in relation to spontaneous pain induced by injection of irritating substances and laparotomy [18]. These changes in facial expression have been associated with painful situations thought to induce negative emotional states [20], thus indicating that rats have the capacity to modify their facial expression in association to emotional states, at least to physiological pain. In mice, some of the facial expressions related to pain have been identified also in aggressive and fearful contexts [19] but no study has looked at rat facial expressions in other than painful situations. To the best of our knowledge no previous research has investigated facial expressions indicative of positive emotional state in rodents. The rat is an ideal model for this investigation since positively valenced behavioural aspects, such as conspecific and heterospecific play, have been extensively studied in this species [21–24], thus allowing for an easier implementation of positive experimental treatments.

The communicative function of facial expressions in rodents is still open to debate. Rats communicate primarily through olfaction [25], touch [26], and vocalisations [27], and only to a minor extent through visual cues [25]. Thus, the adaptive value of facial expressions as a means of social communication could be questioned. Preliminary research in mice however has indicated that lesions of specific brain regions, the activity of which is associated with perceived pain in humans (e.g., the rostral anterior insula; [28]), attenuate facial expressions of pain, suggesting that a face expressing pain may directly reflect the experience of a negative emotional state [20]. This in conjunction with the findings that familiar mice mutually affect their pain behaviour based on each other’s pain status [29] and female mice choose to spend more time near familiar females in pain [30] leaves open the possibility that mice, and perhaps rodents in general, may actually pay attention to the facial expressions of conspecifics to gather information on their emotional state. An alternative adaptive explanation for the function of facial expressions is that individuals may gain self-directed benefits from exhibiting such expressions. In the same way as expressions of fear increase sensory exposure (e.g. widening of the eyes to enhance vigilance) and expressions of disgust decrease it in humans [31], expressions of aggression and/or fear in mice may be aimed at protecting vulnerable parts of the face (e.g., tightening of eyes, flattening of ears; [19]). Although the existence and functions of positive facial expressions in rodents are still unexplored, the same adaptive function hypotheses described for negatively valenced situations could be extended to contexts with positive hedonic value, like play, sex and feeding. During ‘rough-and-tumble’ play, for example, dyads of rats compete for access to each other’s nape using visual and tactile cues to detect and respond to play initiations of their play partner [21]. Considering also that relatively more playful rats have been found to increase play behaviour of their partner, suggestive of social contagion [32,33], it is possible that rats may at least partly sense (via sight and/or touch) the facial expressions of their partner, along with other body postures, to gather information on the likelihood the partner will initiate play, and how intense the play is likely to be. In contrast, similarly to research in humans showing that the unconscious induction of one form of smile contributes to the positive self-assessment of an emotional experience [34], positive facial expressions in rats may *per se* induce a more positive hedonic state (e.g., through relaxation of parts of the face).

Overall, there is general consent on considering positive social interactions, such as play, as rewarding experiences that can elicit positive emotions in animals [35,36]. Conspecific rough-and-tumble play is the most common form of play in juvenile rats [21] and has characteristics that allow it to be clearly distinguished from true fights [22]. In the attempt to mimic this type of play within an heterospecific context between a rat and an experimenter, manual tickling has been developed and studied in relation to the experience of positive emotional state [23,37]. Tickling induces the same positively valenced ultrasonic vocalisations (USVs) that are also emitted during conspecific play [4] and has also been found to increase optimistic judgment bias in rats [24]. Therefore, tickling most likely leads to the experience of positive emotional states, the intensity of which can be determined by measuring the number of USVs emitted by the animals during and between tickling bouts [38].

Rats emit different types of USVs in response to positive and negative situations, thus USVs represent a promising proxy measure of acute emotional valence [4]. Flat 22 kHz calls are emitted when rats experience negative stimuli or during avoidance behaviours [39] and are thus considered to be associated with negative emotional states [27]. On the other hand, frequency modulated (FM) 50 kHz USVs are emitted in situations considered to have an hedonic value, such as in anticipation of and during play, during sexual behaviour, and following stimulation of brain reward pathways [35,40–42], and thus have been used as indicators of acute positive emotional state in rats. Therefore, USVs represent a promising measure to identify rats’ positive or negative emotional states within various experimental conditions including heterospecific play.

The purpose of this study was to determine if rats exhibit facial expressions indicative of positive emotional states after experiencing tickling by an experimenter. Using an explorative approach, a number of qualitative and quantitative measures of facial expressions were scored and analysed using a procedure similar to the one used in already existing rodent grimace scales [18,20]. Measured facial expressions were then compared with those expressed by the same animals in a mildly aversive Contrast Treatment. We hypothesised that if facial expressions reflect inner emotional states, the two different treatments would induce different facial expressions by the same rat.

## Methods

### Ethical statement

This study was conducted in compliance with the Swiss regulations on animal experimentation and formally approved by the Veterinary Office of the Canton of Bern (License no. BE 17/13).

### Animals and housing

**Fig 1:**
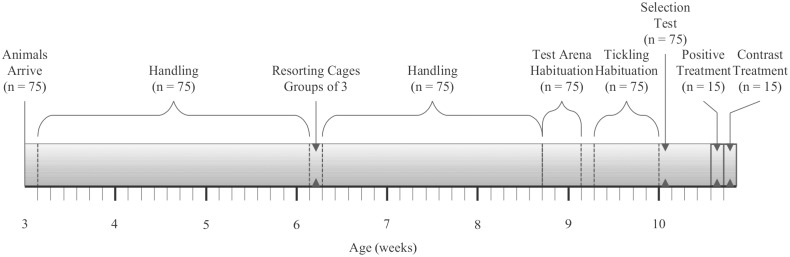
Timeline of experimental design. The timeline includes animal handling, habituation, and experimental treatment phases, indicating number of animals used in each phase.

Subjects were 15 male Lister Hooded rats born from 14 litters. This sample size was based on a power calculation (G*Power, version 3.1.9) with an expected large effect size (Cohen’s d_z_ = 0.8). These animals were selected from a larger pool of 75 rats from 25 litters born at Charles River Laboratories, Sulzfeld, Germany, based on their response to tickling by the experimenter (see "Selection of rats for Positive and Contrast Treatments" section). All 75 rats were transported at weaning (21 ± 1 days of age) to the Division of Animal Welfare, University of Bern, Switzerland, and randomly sorted into groups of four non-littermates. All rats were specific-pathogen-free at arrival, in line with the FELASA recommendations for health monitoring of rodents [43]. After three weeks (43 days of age), all rats were resorted into new groups of three non-littermates, so that each group consisted of individuals with varying levels of conspecific playfulness. This procedure was part of a parallel study investigating the development of social play in relation to individual personality differences [44]. The formed groups remained stable for the duration of the present study, i.e. between 61 and 75 days of age. Animals were housed in "Mickey 2 XL" cages (l × d × h: 80 cm × 50 cm × 38 cm; Savic, Belgium). Cage bedding consisted of wood chips (JRS Lignocel), with three paper towels and three wooden tongue depressors provided weekly as enrichment. Animals had *ad libitum* access to standard rodent food (KLIBA NAFAG, Switzerland) and tap water. Housing room temperature was maintained between 21–24°C and humidity between 50–70%, with a 12:12 h light/dark cycle (lights off at 9 am). All animals were tested during the dark phase and were identified by their pelage patterns. At the end of the study some of the rats were adopted as pets, and the remaining rats were euthanised. For the rats which were euthanised, they were first anaesthetised by means of isoflurane inhalation, and then euthanised through CO_2_ inhalation followed by decapitation. This procedure was in compliance with our experimental licence no. BE 17/13. The experimental design, including subjects and timeline, is shown in Fig 1.

### Test arena design and lighting

The test arena (l × d × h: 40 cm × 50 cm × 40 cm) was made of three sides with an open edge (S1 Fig). Surfaces on the three sides and floor were covered by grey cardstock paper. A grey background was chosen so that both white and black rat whiskers would be visible from the photographs taken. The floor and back wall consisted of one longer piece of paper bent into a concave curve to form an infinity cyclorama. This allowed for photographs to have the most uniform backgrounds possible. In addition, the curved floor encouraged animals to be closer to the open end of the test arena, better allowing for close-up photographs. The microphone for vocalisation recording was suspended 30 cm above the floor of the test arena, allowing for full coverage of the area (see "Recording and analysis of vocalisations" section for recording equipment). The test room was lit by built-in fluorescent white lights on the ceiling and an additional lamp with a 13 Watt bulb was placed adjacent to the test arena. The brightness within the test arena from these two light sources was 650 lux. This brightness was necessary in order to take high-speed photographs (see "Photography, selection, and analysis of images" section). Habituation and Positive Treatment occurred in a test room adjacent to the housing rooms. The Contrast Treatment occurred in a second test room located in close proximity to the housing rooms. Both test rooms had the same test arena design and lighting.

### Habituation to test conditions and tickling procedure

#### Handling

All 75 animals were gently handled in their home cage by two experimenters (K.F. and J.F.L.), with touching, picking up, and some playful tickling. Handling began at 25 days of age (five minutes per cage daily for five weeks) and continued until test arena habituation began.

#### Habituation to test arena and lighting conditions

The experimental design required the animals to be tested under bright light during their active dark phase, thus gradual habituation to increasing periods of isolation, tickling and light exposure was performed in order to overcome anxiety.

All 75 animals experienced an eight day habituation procedure. Throughout the habituation, all three cage mates were removed from the home cage and placed into a standard, transparent Type III cage (Tecniplast, Italy) in the corridor adjacent to the housing rooms, where they were exposed to 350 lux for two minutes to allow for adjustment to light. The two-minute adjustment period was sufficient to prevent the display of any strong behavioural signs of distress (e.g., freezing behaviour, escape attempts) once the rats were placed in the test arena, which was lit at 650 lux.

First, at 61 days of age, all three cage mates were placed together into the test arena for one minute. Then each rat was individually placed in the test arena for two consecutive 30 second periods, while its cage mates were kept in the Type III cage next to the test arena. In the following two days, the isolation time in the test arena was gradually increased to two minutes. On the next three days, all three cage mates were placed together in the test arena and tickled for a three minute period each day. On the last day of habituation each animal was individually brought into the test room, without its cage mates being present in the room, and intermittently tickled for two minutes. At the end of each test arena exposure, all rats were returned immediately to the home cage and rewarded with a piece of sweetened cereal (Honey Loops, Kellogg’s).

### Tickling procedure

**Fig 2:**
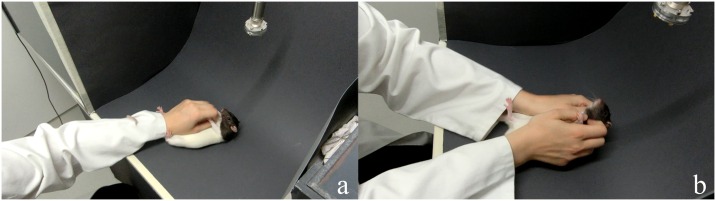
Tickling procedure. The one-handed tickling procedure (a) consisted of one-handed repeated pinning, while rapidly stimulating the underside with the fingertips. The two-handed tickling procedure (b) consisted of scooping and supporting the animal with both hands, while vigorously tickling the sides and nape of the neck with the fingertips.

Animals were habituated to two different tickling procedures in preparation for the Positive Treatment. The first was an established method developed by Panksepp and Burgdorf [38]. It consisted of using one hand to quickly turn the animal on its back and perform rapid, fine-scale finger movements on its neck, chest, and stomach area, then releasing the animal and allowing it to right itself (Fig 2a). Tickling was performed for 3–5 seconds before releasing the animal, and a “tickling bout" consisted of multiple pins, tickles, and releases in a 15 second period. A second, novel tickling procedure was developed by the experimenter to further induce a positive emotional state. Similar to a method used by Rygula et al. [24], where animals were supported on their backs by one hand and tickled with the other, this procedure consisted of holding the rat with two hands and focused more on stimulating the sides and nape of the neck with the fingertips, as the nape is the target of "rough-and-tumble" play in juvenile rats [45]. Rats were scooped with both hands and tilted backwards in supine position. While supported by the hands, the sides and nape of the neck were vigorously jiggled with the fingertips (Fig 2b; see also S1 Appendix for more detail). The FM 50 kHz USVs elicited by this novel method were as loud as or louder than those elicited by the one-handed method (as observed by visual inspection of the spectrograms; S2 Fig). Since one-handed tickling had the advantage of being quite established in the literature, and two-handed tickling had the advantage of producing louder positive USVs, a mixture of both one-handed and two-handed tickling was used in the habituation phase. Within each tickling bout the rat was tickled with two hands once for 3–5 seconds, and tickled with one hand during the remaining bout time.

### Selection of rats for Positive and Contrast Treatments

After the habituation phase, an *ad hoc* selection test was developed and performed to identify the 15 rats (out of the 75 available rats) which responded most positively to tickling, i.e. those that could be considered the most suitable candidates to detect differences between Positive and Contrast Treatments. At 70 days of age, rats were selected following a two-step procedure which consisted of (i) assessing the number of approach behaviours after tickling in the home cage and (ii) counting the number of FM 50 kHZ USVs emitted during tickling in the test arena.

The approach behaviour measure was based on previous research showing that rats which enjoy tickling interactions will pursue the experimenter's hand after a tickling bout [4]. It consisted of performing one-handed tickling for three seconds on each animal in the home cage during the dark phase, repeated three times. The animal had to return to the experimenter's hand or to the open cage door within five seconds, after each of the three tickling events. Resistance to pinning or a slow return served as the exclusion criteria, and those animals were not brought to the second part of the selection test.

Qualifying animals (N = 49) were first habituated to light in groups for two minutes and were then individually placed in the test arena. Over a two minute period, each rat experienced 15 seconds of habituation, followed by four bouts of two-handed tickling, one bout of one-handed tickling, and a final bout of two-handed tickling. The number of positive and negative vocalisations were recorded and analysed (see "Recording and analysis of vocalisations" section). The 15 animals that emitted the greatest number of positive vocalisations and made no negative vocalisations were selected.

### Positive Treatment: Tickling

The Positive Treatment was used to induce a positive emotional state in rats through tickling by an experimenter. At 74 days of age, all animals from each home cage experienced habituation to light for two minutes at 350 lux. Each rat was individually carried by hand into the test room and placed alone in the test arena for two minutes at 650 lux. During the first 15 seconds, the rat was untouched and allowed to acclimatise. Then it received one five second period of two-handed tickling, one of one-handed tickling, and one of two-handed tickling, each separated by a ten second pause. The second minute consisted of rapidly alternating one- and two-handed tickling. It was during the second minute that the experimenter took photographs at close range of the animal (at a maximum distance of 50 cm), focusing on the face. Precisely, photographs were taken immediately after a tickling event was finished and when the animal was upright and facing the camera. Photographs were only taken when positive USVs were emitted. If no positive USVs were heard, the tickling procedure was repeated. After the two testing minutes had passed, the animal was returned to its home cage. During testing, the number and type (positive or negative) of USVs and the presence/absence of faecal boli and urine were recorded. New paper was placed into the test arena before the Positive Treatment phase started. Shed hair was swept between animals in order to maintain a clean background in the photographs.

### Contrast Treatment: Novel room and intermittent white noise

The day after the Positive Treatment, a mildly aversive Contrast Treatment was applied in order to discourage positive USVs and to create contrast photographs to compare to those taken during the Positive Treatment. All equipment was moved to a different, novel test room located in close proximity to the housing rooms. The paper lining was replaced, and lighting and test arena dimensions were recreated to be as similar to the habituation and Positive Treatment test room as possible. Animals were exposed to the novelty of the new room and to intermittent bursts of white noise. White noise was selected as it is a mildly aversive treatment [46] and would not influence the visual quality of the photographs. White noise with a frequency range of 0–22 kHz was used, similar to previous research where predictable bursts of white noise in the 0.05–26 kHz range were used as a stressor on male rats [47].

Each rat was taken directly from the home cage to the second test room in a standard, transparent Type II cage (Tecniplast, Italy). Animals were not gradually habituated to the light of this test room, contrary to the Positive Treatment, in order to make the Contrast Treatment more aversive [46]. Each individual was placed in the test arena and not handled during a two minute period, while intermittent bursts of white noise were played for three seconds every ten seconds. Similar to the Positive Treatment, animals were photographed at close range and immediately after each burst of white noise, throughout the two minute period within the test arena. During testing, the number and type (positive or negative) of USVs and the presence / absence of faecal boli and urine were recorded. Shed hair was swept between animals. Testing order was kept the same within Positive and Contrast treatments so that residual odours from previously tested rats remained similar across treatments for each animal.

The order of Positive and Contrast Treatments was not counterbalanced across animals. Although the effects of treatment and day of treatment were confounded, this experimental design presented two advantages. First, it ensured that the rats only had positive associations with the test arena until and during the Positive Treatment. As the test arena used was the same for both Positive and Contrast Treatments, exposing animals to the Contrast Treatment first might have negatively affected the emotional state of rats in the Positive Treatment due to carry-over of the mildly aversive experience in the test arena during the Contrast Treatment. Instead, the Positive Treatment was always performed first, following a gradual habituation to the tickling procedure which ensured positive anticipation across animals at testing during the Positive Treatment. Second, the aim of the study was to induce the desired emotional state within each treatment, and to confirm this, USVs were recorded during both treatments. Thus, it did not matter whether the effect of day contributed to and/or influenced the emission of USVs, as long as the comparison of USVs between treatments confirmed that the Positive Treatment induced a positive emotional state, and the Contrast Treatment a non-positive to mildly negative emotional state.

### Recording and analysis of vocalisations

Vocalisations were recorded with the Avisoft-UltraSoundGate 116Hb recorder and a high quality condenser microphone (Avisoft Bioacoustics, Germany). Recordings had a frequency range of 5 to 120 kHz, with a sampling rate of 250 kHz and 16 bit resolution. During habituation and testing, USVs were made audible to the experimenter through a real-time transformation of frequencies (under-sampling of 10:1).

Vocalisations were counted from spectrograms using the Avisoft-SASLab Pro software (Avisoft Bioacoustics, Germany). Following Wright et al. [48], spectrograms were created with a fast Fourier transform (FFT) length of 512 points and overlap of 75% (FlatTop window, 100% frame size). In order to be counted as separate vocalisations, USVs had to be at least 20 milliseconds apart [48]. Positive vocalizations were defined as FM 50 kHz calls containing a trill component, regardless of whether they contained step, flat, ramp, or jump components [48]. Trills consisted of at least two "inverted-Us" of rapid frequency oscillations within a period of five milliseconds [48]. Negative vocalisations were defined as vocalisations with near-constant frequency ranging from 20 to 25 kHz and lasting from 200 to 2000 ms [48,49].

### Photography, selection and analysis of images

In previous studies of rodent facial expression, frames showing the face were grabbed either manually [20] or automatically [18] from video recordings. This method was considered unsuitable for the current experimental design, as the quality of video frames would be insufficient for detecting more subtle differences. Additionally, the tickling procedure allowed the animal to move across a greater space and depth than in previous studies, which made filming from a fixed location impractical.

Therefore, we used a hand-held photo camera (Casio Exilim HS EX-ZR800) set to a high-speed mode capable of capturing up to 30 photographs per second. This shutter speed requires relatively high light levels in order to focus and capture the images correctly, hence the need to have bright lighting within the test arena. A flash was not used.

**Fig 3:**
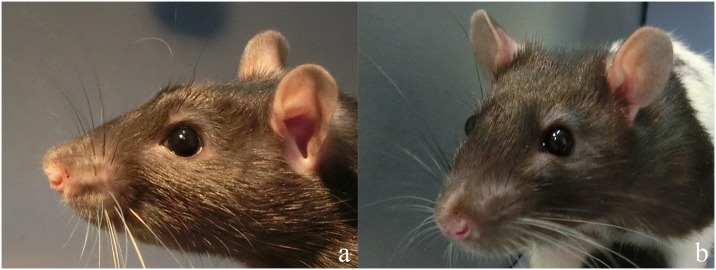
Examples of profile (a) and quarter (b) images. Profile images were selected for the assessment of quantitative measures as they allowed to detect more subtle differences across animals and treatments. Quarter images were used for qualitative measures as they showed more of the face and their assessment was only minimally affected by the head pointing upwards or downwards.

About 100–150 photographs were taken per animal per treatment, creating an available pool of about 3000 images. Images were discarded if they did not show the face or were blurry. Moreover, only images of clear profiles (Fig 3a) and quarter (three-quarter) angles (Fig 3b) were selected for the analysis. Profile angle images allowed for comparisons of quantitative measures. Quarter angle images were used for qualitative scoring, as they showed more areas of the face and appeared more standardised (i.e., from this angle the assessment of the face was minimally affected by the head pointing upwards or downwards) than the images selected in previous rodent grimace scales [18].

Four Profile angle images per treatment and four Quarter angle images per treatment were randomly selected for each rat (with the exception of two animals which had only two acceptable images within one Positive and one Contrast Treatment), resulting in 236 images in total to score (15 rats × two treatments × two photograph angles). Randomisation was performed by generating a random sequence of images for each rat, treatment and photograph angle (www.random.org), and by selecting the first four images of this sequence. All selected images were cropped in CorelDRAW X7 (64-bit) to only show the head and as little of the body as possible to avoid potential biases during image analysis. All images were randomized and relabelled by a separate experimenter for blinded scoring.

### Selection of facial expression measures

The measures taken for image analysis are described in Table 1. Qualitative measures, taken from quarter images, included established measures from the Rat Grimace Scale [18] and two novel measures, namely the visibility of the Nictitating Membrane and the intensity of the pink Ear Colour.

**Table 1:** Qualitative and Quantitative measures taken for image analysis.

**Qualitative—Quarter Images**	
Orbital Tightening*	A narrowing of the orbital area, as partial/complete closure
Nose/Cheek Flattening*	A flattening between the cheek and whisker pads
Ear Change*	Ears become curled and pointed in shape
Whisker Change*	Whiskers move away from the face and tend to bunch
Ear Colour	Ears become pinker and more flushed
Nictitating Membrane	Increased visibility of the membrane at the front of the eye
**Quantitative—Profile Images**	
Eye Ratio	Eyeball height divided by eyeball width
Eyebrow Ratio	Height from the bottom of the eyeball to the brow ridge, divided by eyeball width
Eyebrow Angle	Measure of the openness of the eye via eyebrow angle
Ear Angle	Measure of the position of the tilted ear along the face

* Measures from Rat Grimace Scale [18].

The Rat Grimace Scale was used to assess whether the “Action Units” associated with the expression of pain [18] were also affected by the states induced in the Positive and/or the Contrast treatments. The Nictitating Membrane or "third eyelid" is a membrane that protects the cornea [50]. It is occasionally visible in Rat Grimace Scale images but has not been specifically assessed previously; hence it was added as a separate measure. The Ear Colour measure was added based on preliminary observations of the animals during the habituation phase indicating that the colour intensity of the ears appeared to change when the animals were habituated to tickling.

Quantitative measures, taken from profile images, included measures of the width and height of the eye and eyebrow, and angles of the ear and eyebrow. In rodents, the eyes and ears have been shown to be particularly expressive areas of the face, changing dramatically in negatively valenced situations [18,19], yet it is unknown whether these can also be affected by positively valenced situation. Therefore, by measuring these areas of the face quantitatively, we aimed to detect more subtle changes compared to qualitative assessments.

### Qualitative measures

The facial regions which were scored based on the Rat Grimace Scale included Orbital Tightening, Nose/Cheek Flattening, Ear Change, and Whisker Change (scores between 0–2 for each facial region; see [18]).

Furthermore, two novel qualitative measures were taken, namely the variation in intensity of the ears’ pink colour and the visibility of the Nictitating Membrane. These were scored on the same three point scale as the Rat Grimace Scale measures.

**Fig 4:**
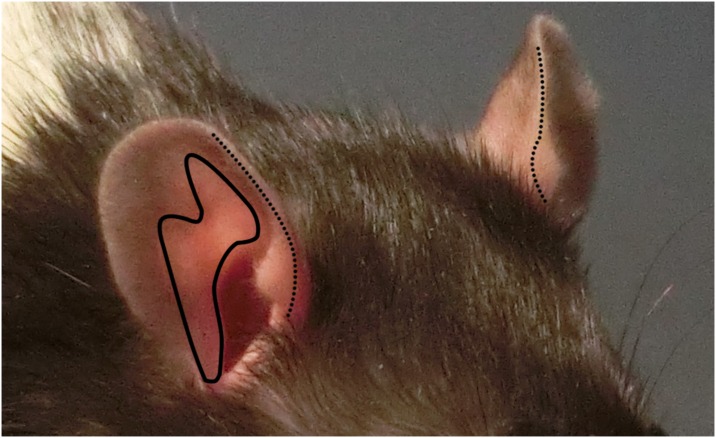
Areas of the ear to score for Ear Colour from quarter images. Ear colour should be determined by looking at the leading edge of the ear flap (dotted line) and lighter skin on the interior side of the flap (solid line). Both ears should be observed for colour, with preference given to the more visible ear. The ear canal, if visible, should not be used to determine colour, and shadows within folded over ears need to be considered as they may darken the skin inside the ear.

Ear Colour was scored by judging the paleness or “pinkness” of the skin on the external flap of the ear. Only specific areas of the ear, the pigment-free inner skin and leading edge of each ear were used for scoring (Fig 4). As this measure was novel, practice scorings were carried out and scoring guides prepared (see S2 Appendix).

Variation in overall image colour occurred due to the nature of the high-speed photography. Although care was taken to maintain identical lighting conditions between the two treatment rooms, images from the Contrast treatment were darker than those from the Positive treatment, possibly due to animals in the Positive Treatment spending more time close to the edge of the test arena (i.e., closer to the camera) after tickling bouts. As this could potentially affect Ear Colour scoring, images classified as too dark were not included in the analysis, and the remaining images were colour corrected using Microsoft Office Picture Manager (2006 Microsoft Corporation). All images were edited in order to have uniform white balance, which allowed to minimise the influence of temperature differences (variation in colour along the blue-yellow axis) across images on scoring [51].

The visibility of the Nictitating Membrane was scored by judging how visible the white-coloured nictitating membrane was at the front of the eye (see S3 Appendix for scoring guide).

### Quantitative measures

**Fig 5:**
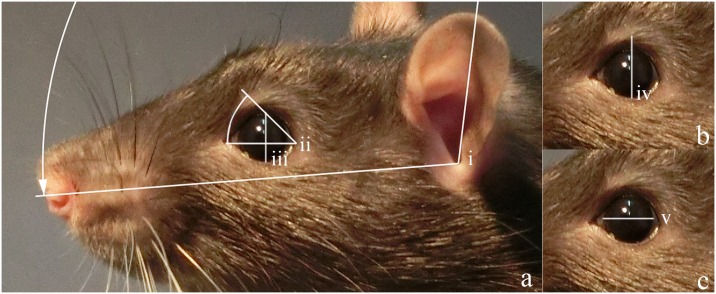
Examples of quantitative measures. a) Ear Angle (i), Eyebrow Angle (ii), and eye height (iii) are illustrated. b) Eyebrow height (iv) is measured from the bottom of the eyeball to the middle of the brow. c) Eye width (v) was the diameter of the visible iris.

Quantitative measures were taken in CorelDRAW X7 using the "Parallel Dimension" tool to measure eye width and height in millimetres and the "Angular Dimension" tool to measure Eyebrow and Ear Angle (Fig 5). Eye width was the diameter of the iris, measured across the horizontal centre of the eye (Fig 5c, measure v). Eye height was the distance from the bottom to the top of the iris (Fig 5a, measure iii). Eye Ratio was eye height divided by eye width to detect a change in eye openness. Eyebrow height was measured from the bottom of the eyeball to the middle of the lightly furred "brow" above the eye (Fig 5b, measure iv). Eyebrow Ratio was the result of eyebrow height divided by eye width to detect a raising of the brow. Ratios allowed for consistent measurements across images cropped to different sizes. Therefore, only the calculated ratios were analysed.

Eyebrow Angle was the angle between the line connecting the back of the eye, midline of the eye and the tear duct, and the line connecting the back of the eye to the forward point of the eyebrow (Fig 5a, measure ii). This point is identified by the most forward placed whisker on the brow (visible when magnified in program). Ear Angle was the angle between the line connecting the bottom of the ear canal to the tip of the nostril and the line connecting the bottom of the ear canal to the tip of the ear (Fig 5a, measure i).

### Intra- and Inter-rater reliability

For all measures, intra-observer reliability was assessed by rescoring every fifth image (20% of all images) by the same experimenter (K.F.). Quantitative and qualitative measures that showed a significant treatment effect were rescored (all images) by a second experimenter (J.L.) for the assessment of inter-rater reliability.

Categorical data (qualitative measures) reliability was assessed using Cohen’s Kappa tests. Only reliability coefficients greater than 0.6 (indicating “substantial” to “perfect” agreement; [52]) were considered acceptable. Continuous data (quantitative measures) reliability was measured using intraclass correlation (ICC). ICC was calculated using a two-way mixed design assessing the absolute agreement of the mean of the ratings [53,54]. Only ICC coefficients greater than 0.6 (indicating “good” to “excellent” agreement; [55], and with a lower bound of the 95% confidence interval (CI) greater than 0.5, were considered to be acceptable.

If either Cohen’s Kappa or ICC coefficients were lower than 0.6, the rater was retrained with scoring guides and testing for reliability was repeated.

### Data analysis

Statistical analyses were performed using R (version 3.2.1.) and SPSS (version 22). Ordered logistic regressions and linear mixed-effects models were used for the analysis of qualitative and quantitative measures, respectively. To adequately reflect dependencies in the experimental design, all models had treatment as fixed effect, while random effects were image nested in treatment nested in rat. Model assumptions were assessed by graphical inspection of the residuals. We verified normal distribution and homogeneity of variance for the linear models, and homogeneity of variance for the ordered logistic regressions. Treatment effect on positive USV frequencies was assessed using a paired t-test. Transformation of the data was not necessary for any of the models used.

The measures Orbital Tightening, Nose/Cheek Flattening, Whisker Change, Ear Change, negative USV frequencies, faecal boli frequencies and presence/absence of urination had only (or almost only for Ear Change) "0" scores / frequencies in both treatments, thus only a descriptive analysis was performed for these measures. Familywise error rate resulting from the analysis of the seven remaining outcome measures was controlled for using Bonferroni correction, after which the threshold p value for significance was set to 0.007. This correction, being a more conservative approach against familywise error rate, ensured that any significant findings would most likely represent true differences.

Data are presented as score percentages for the qualitative measures, and as Mean (*M*) and Standard Error of the Mean (*SEM*) for the quantitative measures and positive USV frequencies.

## Results

### Validity of Positive and Contrast Treatments

**Fig 6:**
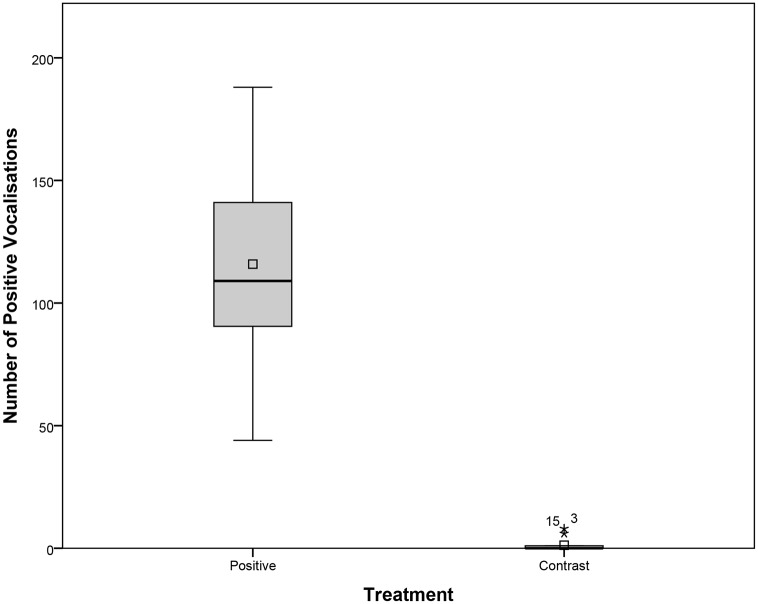
Boxplot showing the frequencies of positive USVs emitted by the rats during Positive and Contrast Treatments. The box represents the middle 50% of the data, while the upper and lower whiskers show the extreme data points, excluding outliers (starred dots). The horizontal line within the box represents the median, while the square dot shows the mean.

Rats emitted a higher number of positive USVs in the Positive Treatment (*M* = 115.87, *SEM* = 10.29, range: 44–188) compared to the Contrast Treatment (*M* = 1.07, *SEM* = 0.63, range: 0–8; *t*(14) = - 10.96, *p* < 0.001; Fig 6). Negative vocalisations, faecal boli, or urination did not occur in any of the treatments.

### Intra-rater reliability of qualitative and quantitative measures

Cohen’s Kappa tests assessing intra-rater reliability of qualitative measures showed that there was perfect agreement for Orbital Tightening, Nose/Cheek Flattening, and Whisker Change measures (*κ* = 1.00) and substantial agreement for Nictitating Membrane visibility (*κ* = 0.76) and Ear Colour (*κ* = 0.80, *p* < 0.001). Reliability was found not to be acceptable for Ear Change (*κ* = 0.22). The rater was therefore retrained, and after a second rescoring of all images for Ear Change, reliability improved to perfect agreement (*κ* = 1.00).

Intra-rater reliability of the quantitative measures as assessed by intraclass correlation coefficient was good for Eyebrow Angle (ICC_average_ = 0.80; CI lower bound = 0.54) and excellent for eyeball width (ICC_average_ = 0.99; CI lower bound = 0.98), eyeball height (ICC_average_ = 0.99; CI lower bound = 0.98), eyebrow height (ICC_average_ = 0.99; CI lower bound = 0.98), and Ear Angle (ICC_average_ = 1.00; CI lower bound = 1.00).

### Treatment effects—Qualitative measures

All statistics for the qualitative measures are summarised in Table 2.

**Table 2:** Summary of descriptive and inferential statistics for the qualitative measures.

Outcome measure	Model	Scores	Proportions [%]		Test statistic	P-value ^1^
			Positive	Contrast		
Orbital Tightening	Descriptive analysis	0	100	100		
		1	0	0	n/a	n/a
		2	0	0		
Nose/Cheek Flattening	Descriptive analysis	0	100	100		
		1	0	0	n/a	n/a
		2	0	0		
Whisker Change	Descriptive analysis	0	100	100		
		1	0	0	n/a	n/a
		2	0	0		
Ear Change	Descriptive analysis	0	100	86.2		
		1	0	10.3	n/a	n/a
		2	0	3.4		
Ear Colour	Ordered logistic regression	0	3.3	53.4		
		1	25.0	39.7	*χ*^2^_1_ = 32.04	< 0.001 *
		2	71.7	6.9		
Nictitating Membrane	Ordered logistic regression	0	40.0	55.2		
		1	48.3	36.2	*χ*^2^_1_ = 0.52	0.47
		2	11.7	8.6		

^1^ Critical p-value after Bonferroni correction for multiple hypotheses testing: p = 0.007.

* Denotes significant difference.

#### Rat Grimace Scale measures

Orbital Tightening, Nose/Cheek Flattening, and Whisker Change measures had only "0" scores in both treatments. Similarly, Ear Change measure had only "0" scores, with the exception of six "1" scores and two "2" scores in the Contrast Treatment. Therefore, these measures were not further analysed.

#### Ear Colour and Nictitating Membrane

**Fig 7:**
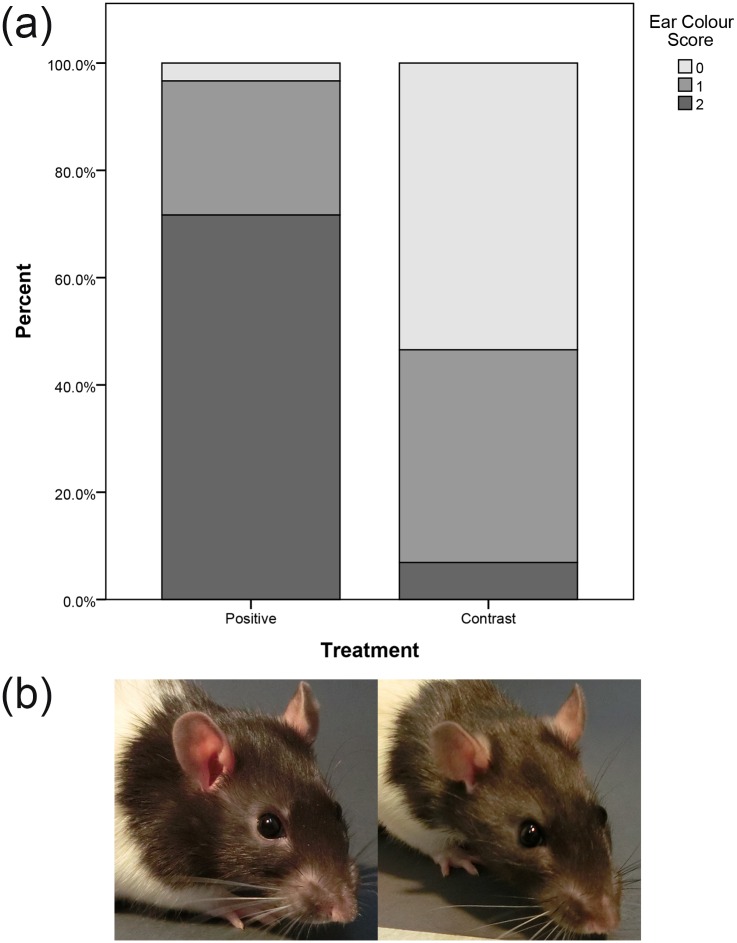
a) Stacked bar chart showing Ear Colour score percentages within Positive and Contrast Treatments. A score of 0 corresponds to “not present”, a score of 1 to “moderately present” and a score of 2 to “obviously present”. b) Example images representative of Ear Colour in the Positive (left) and Contrast (right) Treatments.

Ear Colour scores were significantly higher in the Positive Treatment than in the Contrast Treatment (*χ*^2^_1_ = 32.04, *p* < 0.001; Fig 7). The Nictitating Membrane visibility scores did not vary significantly between Positive and Contrast Treatments (*χ*^2^_1_ = 0.52, *p* = 0.47).

### Treatment effects—Quantitative measures

All statistics for the quantitative measures are summarised in Table 3.

**Table 3:** Summary of inferential statistics for the quantitative measures.

Outcome measure	Model	Mean ± SEM		Test statistic	P-value ^1^
		Positive	Contrast		
Eye Ratio	Linear mixed-effects model	0.933 ± 0.012	0.941 ± 0.0089	*F*_1,14_ = 0.39	0.54
Eyebrow Ratio	Linear mixed-effects model	1.28 ± 0.012	1.29 ± 0.013	*F*_1,14_ = 0.29	0.60
Eyebrow Angle	Linear mixed-effects model	41.1° ± 0.52	41.1° ± 0.36	*F*_1,14_ = 0.001	0.98
Ear Angle	Linear mixed-effects model	127° ± 1.3	112° ± 1.5	*F*_1,14_ = 19.66	< 0.001 *

^1^ Critical p-value after Bonferroni correction for multiple hypotheses testing: p = 0.007.

* Denotes significant difference.

**Fig 8:**
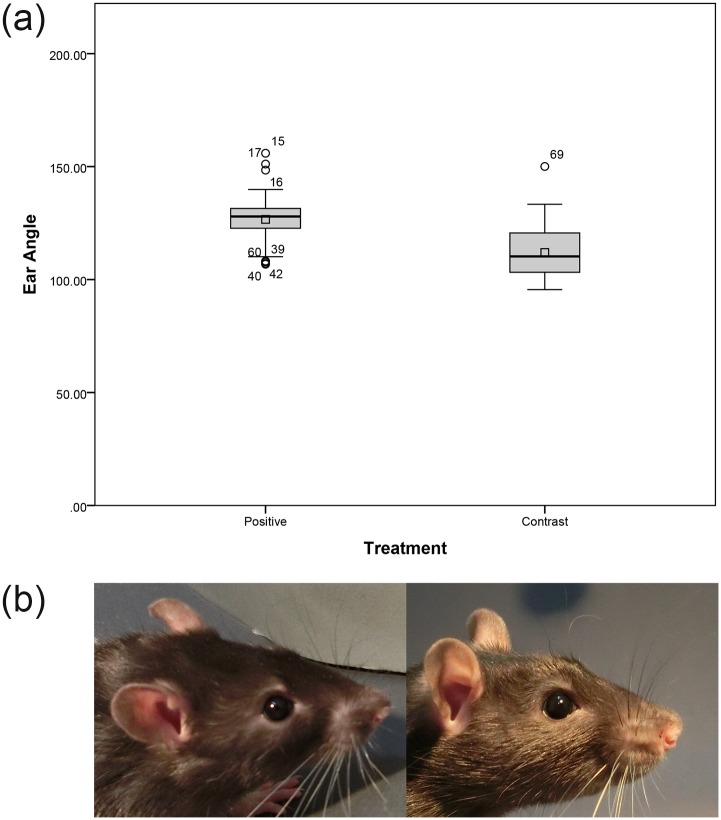
a) Boxplot showing the Ear Angle of rats during Positive and Contrast Treatments. The box represents the middle 50% of the data, while the upper and lower whiskers show the extreme data points, excluding outliers (starred dots). The horizontal line within the box represents the median, while the square dot shows the mean. b) Example images representative of Ear Angle in the Positive (left) and Contrast (right) Treatments.

There was no significant difference between Contrast and Positive Treatments for Eye Ratio (*F*_1,14_ = 0.39, *p* = 0.54) and Eyebrow Ratio (*F*_1,14_ = 0.29, *p* = 0.60). Eyebrow Angle also did not significantly differ between Contrast and Positive Treatments (*F*_1,14_ = 0.001, *p* = 0.98). Ear Angle was significantly wider in the Positive Treatment than in the Contrast Treatment (*F*_1,14_ = 19.66, *p* < 0.001; Fig 8).

### Inter-rater reliability for Ear Colour and Ear Angle

Cohen’s Kappa test showed substantial inter-rater agreement for Ear Colour (*κ* = 0.73). However, inter-rater reliability for Ear Angle was not acceptable (ICC_average_ = 0.80; CI lower bound = -0.17), thus the rater with slightly poorer intra-rater reliability for this measure (J.L.; ICC_average_ = 0.99; CI lower bound = 0.98) rescored all profile images a second time after an additional session of training. The inter-rater reliability for Ear Angle after rescoring was excellent (ICC_average_ = 0.98; CI lower bound = 0.96).

## Discussion

The aim of this study was to determine if rats exhibit facial expressions indicative of a positive emotional state after experiencing tickling by an experimenter. The experimental treatments appeared to have induced the desired positive emotional state, as shown by the greater number of positive vocalisations emitted in the Positive compared to the Contrast Treatment. The main findings were that Ear Colour became significantly pinker in the Positive Treatment and Ear Angle was wider in the Positive Treatment, while all other measures of facial expression did not show differences between treatments. Furthermore, methodological improvements were made in the photography technique and tickling procedure.

### Validity of experimental treatments

The aim of the two experimental treatments was to induce two different emotional states in order to detect differences in facial expression. The Positive Treatment aimed at inducing an acute positive emotional state while the Contrast Treatment was performed to ensure the animals experienced a non-positive to mildly negative emotional state.

Rats emit FM 50 kHz USVs almost exclusively in situations having a hedonic value [35,40–42]. All rats emitted a high number of these USVs in the Positive Treatment, while they made few or no positive USVs in the Contrast Treatment.

During the tickling procedure, rats emit positive vocalisations both while being tickled and during pauses between tickling bouts, and the number of positive USVs emitted in these two phases of the tickling procedure are positively correlated [56]. In this study, rats were photographed during pauses between tickling bouts and only while they were emitting positive USVs, to ensure that the hedonic effect of tickling could be captured in the photographs without any influence from the tickling manipulation by the experimenter.

Indicators of severe anxiety and stress were absent during both treatments. Animals did not produce any 22 kHz USVs indicative of negative emotional state [39], nor did they produce faecal boli or urinate [57], thus both treatments were not strongly aversive.

Therefore, it can be concluded that the Positive Treatment was successful in inducing a positive emotional state, and the Contrast Treatment was successful in inducing a more neutral, non-positive emotional state.

### Qualitative measures

Qualitative scoring was comprised of the established Rat Grimace Scale [18] together with two novel measures, Ear Colour and the visibility of the Nictitating Membrane. We found that Ear Colour was significantly pinker in the Positive compared to the Contrast Treatment, while the Rat Grimace Scale measures and the Nictitating Membrane visibility were not affected by treatments.

Images of animals in the Positive Treatment were rarely scored as 0 (not present) for Ear Colour, while the majority of them were scored as 2 (obviously present). This indicates that to a certain degree all rats in the study had ears showing a flushed, pink colour as a result of the Positive Treatment (yet see potential limitations of image assessment in Photography section).

At normal body temperature (37°C), rat ears appear "pale" in colour, with faintly visible blood vessels [58]. Conversely, ears appear "pinker" as a consequence of an increased dilatation of vessels in the skin [59]. This vasodilatation could stem from a variety of conditions occurring in the Positive Treatment.

First, blood vessels within the rat ear dilate when "the ear itself […] is rubbed between the fingers" ([58], p. 313). Therefore, the occasional physical stimulation of the ears against the floor of the test arena or fingertips during the tickling procedure could have increased vasodilatation. However, the ears were at no point the target of the tickling procedure, thus it appears unlikely that the pinker colour observed in the Positive Treatment was mainly or exclusively caused by physical stimulation of the ears.

Second, the physical exertion of tickling may have contributed to an increase in heart rate. Locomotor activity positively correlates with heart rate in the rat [60], and increased heart rate is linked to increased vasodilatation in the body [61], which may have induced the change in ear colour. Thus, differences in physical activity between treatments could explain Ear Colour change.

Lastly, besides physical activity, heart rate and thus vasodilatation can also increase due to (positive) emotional arousal. For example, when conditioned to anticipate a food reward, rats show a significant increase in heart rate while consuming the reward [62]. Although heart rate is mainly a measure of emotional arousal rather than valence [63,64], the increased vasodilatation within the ears together with the simultaneous emission of FM 50 kHz vocalisations may reflect physiological changes in the body indicative of a positive emotional state.

The Rat Grimace Scale measures showed no differences between treatments. In both treatments rats exhibited very few or none of the facial expressions indicative of pain, i.e. Orbital Tightening, Nose/Cheek Flattening, Ear Change, and Whisker Change. This result supports the conclusion that these grimacing expressions are related to pain [18] or potentially associated with negative emotional state more generally as demonstrated in mice [19], but that they are not expressed when rats experience positive emotional states. In addition, the Rat Grimace Scale measures were minimally affected by the Contrast Treatment which confirms that this treatment was not strongly aversive to the animals. Finally, since the rats were not squinting during treatments (all Orbital Tightening scores were 0) we can conclude that the lighting conditions were not highly aversive and that the animals had adjusted to the brightness before photographs were taken.

The Nictitating Membrane was only moderately to not visible in either treatment. An increased visibility of the Nictitating Membrane can be interpreted as a non-specific sign of illness and/or injury in dogs and cats [65], and has been linked to pain-related orbital tightening in rats [18]. It is therefore possible that the visibility of this membrane may be specific to pain-related conditions of illness or injury, which would explain the absence of a treatment effect in our study. Moreover, the visibility of the membrane may be influenced by the direction in which the rat is looking. This may limit the practicability of this measure since only images with rats looking in the same direction should be compared with each other.

### Quantitative measures

Quantitative measures were used to identify subtle changes in facial expression that could not be easily detected by eye.

In the Contrast Treatment, ears were orientated forward and had smaller angles compared to the Positive Treatment, during which the ear angle was wider, with the ears angled more to the side and the back. Previous research has shown that specific changes in the rats’ ear position (ears angled forward or outward) are also indicative of pain [18]. While a similarly wide ear angle could be observed both in the Positive Treatment in this study and after an acute pain treatment [18], the ears of rats in acute pain also tended to fold and curl [18] which we did not observe in our Positive Treatment. In addition, resident mice in a resident-intruder test showed a flattened ear position [19], which again was not shown in the Positive Treatment. Therefore, the current study suggests that in rats changes in ear position can occur also in relation to positive emotional states and can be differentiated from those following a negative event. Similarly, Proctor and Carder [66] found that dairy cows either held their ears backwards or let them hang loosely when being stroked, a treatment that aimed to induce a low arousal, positive emotional state. Furthermore, sheep let their ears hang loosely during positive feeding situations [67] and held their ears horizontally along the frontal plane of the head in neutral, non-negative situations [68]. Despite the differences in the anatomy of the ear among the above-mentioned species, it appears that ears held to the side and let hang loosely downwards and/or backwards (without any folding and curling) may represent a reliable indicator of positive (or at least non-negative) emotional state across a number of animal species. Moreover, this ear position has been observed in both a low arousal situation (e.g. during stroking; [66]) and a high arousal situation such as tickling, thus it appears more likely to be a measure of (positive) emotional valence rather than emotional arousal.

The relatively more pricked ears observed in the Contrast Treatment may be indicative of an alert state most likely induced by the white noise and/or the novelty of the test room. Rats exhibit pricked ears when restrained [69], and when exposed to unpredictable white noise, they show a startled reaction and rear to listen to the sound [47]. Both restraint and sudden noise can be considered aversive stimuli [47,69], which may lead to the experience of negative emotional states [70]. Similarly, sheep spent a higher proportion of time orientating their ears forward while socially isolated [67]. Thus it may be that the pricked ears observed in the Contrast Treatment reflected a relatively more tense and/or anxious emotional state compared to the Positive Treatment, although the Contrast Treatment appeared to be only mildly aversive as the rats produced no faecal boli or negative USVs.

The other quantitative measures of eyebrow angle and eye openness showed no significant differences between treatments. While rats can narrow their eyes when in pain [18], the eye did not open wider or narrow in the Positive compared to the Contrast Treatment. Therefore, within our experimental settings, eye openness immediately following a tickling bout did not seem to be associated with positive emotional state.

### Improvements to photography technique and tickling procedure

#### Photography

A hand-held photo camera was used for capturing facial expressions in both experimental treatments. Compared to video cameras that were fixed in their position in other studies on facial expressions [18,20], holding the camera by hand in this study allowed the experimenter to follow the rats’ movements and adapt the angle of the camera to the position of their faces. Moreover, since pictures were taken with the photo camera and not by frame grabbing from video clips, picture quality was very high, making the identification of subtle differences in the face possible. However, high-speed photography is vulnerable to variation in photograph brightness. Despite attempts to maintain identical lighting within both test rooms, photographs taken during the Contrast Treatment were more likely to be darker than those taken during the Positive Treatment, possibly because the animals used different areas of the test arena during the two treatments. This difference in brightness may have an influence on colour-based scoring systems (i.e. Ear Colour), and images that are too different in brightness should be removed from the analysis and / or colour-corrected to avoid scoring biases, as done in this study. Additional tools assessing variation in blood flow, such as infrared thermography [71] could also be used in conjunction with Ear Colour scoring.

Furthermore, a relatively high light intensity (650 lux) was required to obtain good quality images, and this could have potentially affected the response of the rats to the treatments. However, the light intensity used in this study falls within the range of laboratory room light conditions [25], and the rats were gradually and extensively habituated to being tickled under white light. Since, overall, the rats emitted a large number of positive USVs during the Positive Treatment, we conclude that light intensity at testing is unlikely to have affected our results.

#### Novel tickling procedure

Tickling in previous studies consisted of either repeatedly pinning the rat to a surface with one hand [38] or holding the rat with one hand, while tickling with the other [24]. In addition to the method developed by Panksepp and Burgdorf [38], this study used a novel procedure consisting of scooping the rats with both hands and vigorously jiggling the sides and the nape of the neck.

Animals appeared to respond more positively (e.g., with louder positive USVs as assessed by visual inspection of spectrograms; S2 Fig) when handled using the novel tickling procedure. Due to the relatively hard floor of the test arena, the scooping-and-tilting backwards may have been more comfortable for the rat compared to a flip-and-pin onto the floor. Rats in this study were older and heavier compared to previous studies [4,38], so this method may be more appropriate for older animals. Furthermore, this novel tickling procedure entails greater stimulation of the nape, which is the main target in rough-and-tumble play in rats [21], thus it may be experienced by the rat as more similar to conspecific play.

### Conclusions and further research

The identification of positive facial expressions indicative of positive emotional states in rats could serve as an alternative to or complementary part of existing methods (e.g., positive USVs, cognitive bias) to assess positive animal welfare. This study identified two potential, preliminary indicators of positive facial expression in rats, namely change in Ear Colour and Ear Angle, by comparing a positive emotional state induced by playful tickling and a neutral to mildly negative emotional state induced by exposure to a novel environment and white noise. Even though potentially being limited to the specific treatments and image assessment methods in this study, these measures could provide a basis for future research on positive emotional states. Changes in Ear Colour are unlikely to support any communicative function between rats, as rats have poor colour discrimination and are more active in darkness [25], but may rather reflect the experience of internal physiological changes. Similarly, changes in Ear Angle are likely to indicate relaxation of the muscles controlling ear movement during a positive experience [66,67] which cost less energy compared to situations requiring alertness.

To improve generalisability of our findings, further work on Ear Colour should focus on disentangling positive emotional valence from the physical activity of tickling. For example, having a control treatment in which the neck and dorsal spine of the rat are physically stimulated in order to induce negative 22 kHz USVs (e.g., [72]), could serve as a way to measure emotional valence while controlling for arousal and somatosensory stimulation. Also, further research should investigate the change in ear posture and colour in relation to a variety of positive and negative stimuli other than tickling and unpredictable white noise, respectively. For instance, rats presented with rewarding gustatory stimuli performed rhythmic tongue movements and changes in facial musculature [73], resembling facial expressions made by non-human primates and human infants when exposed to similar flavours [11]. It would be interesting to investigate whether such tongue protrusions are also associated with changes in ear angle and colour. With respect to changes in Ear Angle, it would also be interesting to measure the frequency of changes in ear position and the asymmetry in ear posture through continuous focal observations [67,68].

## Supporting Information

S1 Appendix Novel Two-Handed Tickling Method. (DOCX)

S2 Appendix Ear Colour Scoring Guide. (DOCX)

S3 Appendix Visibility of the Nictitating Membrane Scoring Guide. (DOCX)

S1 Dataset Dataset. (XLSX)

S1 Fig Test arena set-up. (DOCX)

S2 Fig Spectrograms of positive USVs during one-handed and two-handed tickling. (DOCX)
