# Supplementary material for: Facial Indicators of Positive Emotions in Rats
Source: PLoS One. 2016 Nov 30;11(11):e0166446. doi: 10.1371/journal.pone.0166446 (PMC5130214; doi:10.1371/journal.pone.0166446)

**S1 Appendix. Novel Two-Handed Tickling Method.**

**Part 1:** The rat faces the experimenter. It is scooped with both hands, by placing the thumbs under the forearms and remaining fingers around the shoulders and down the back. It is gently but quickly tilted onto its back.


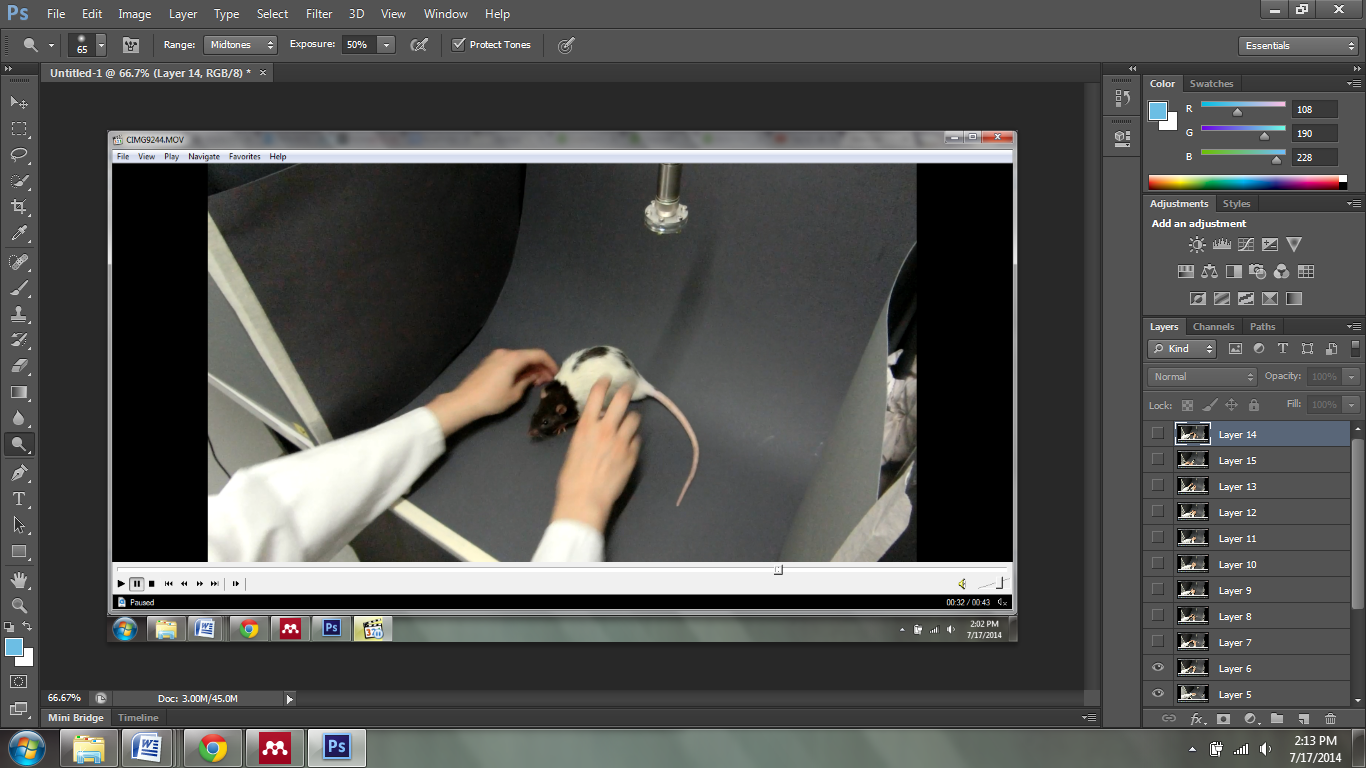

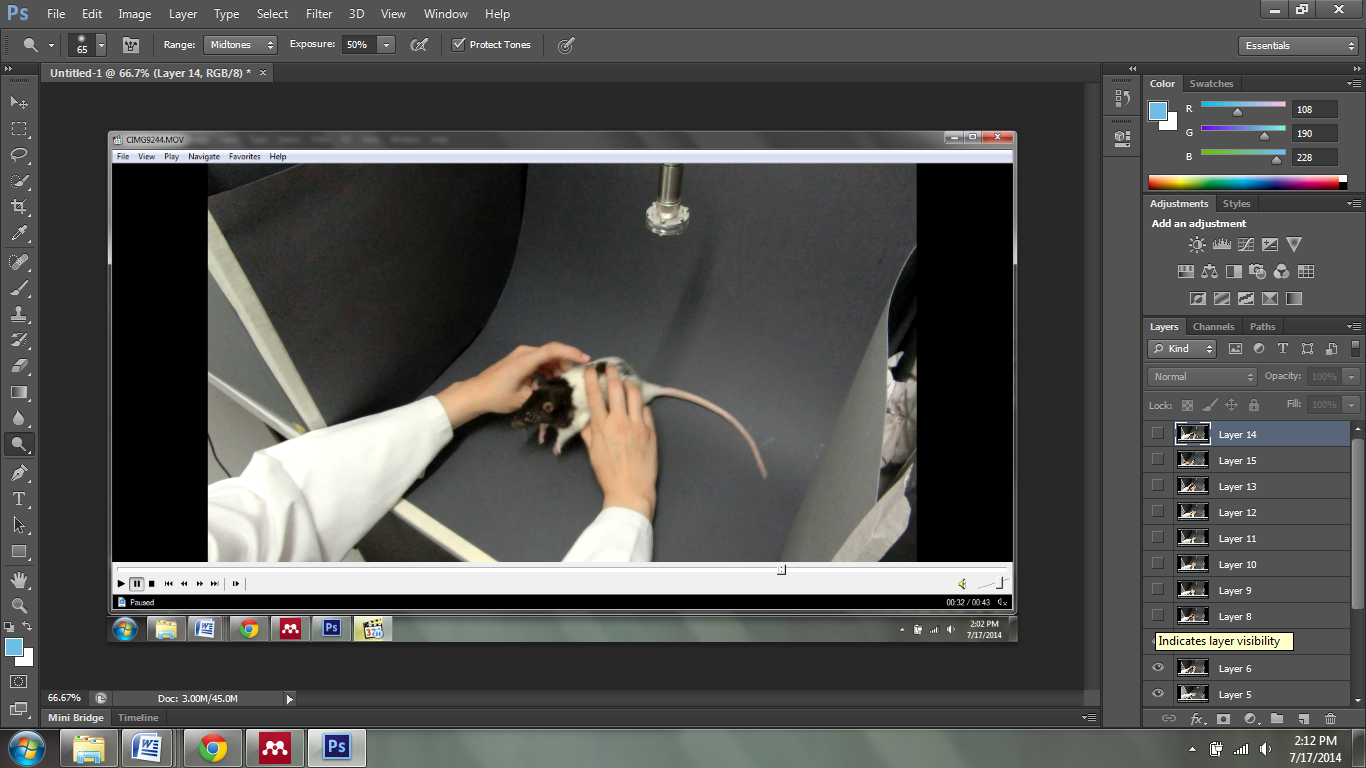

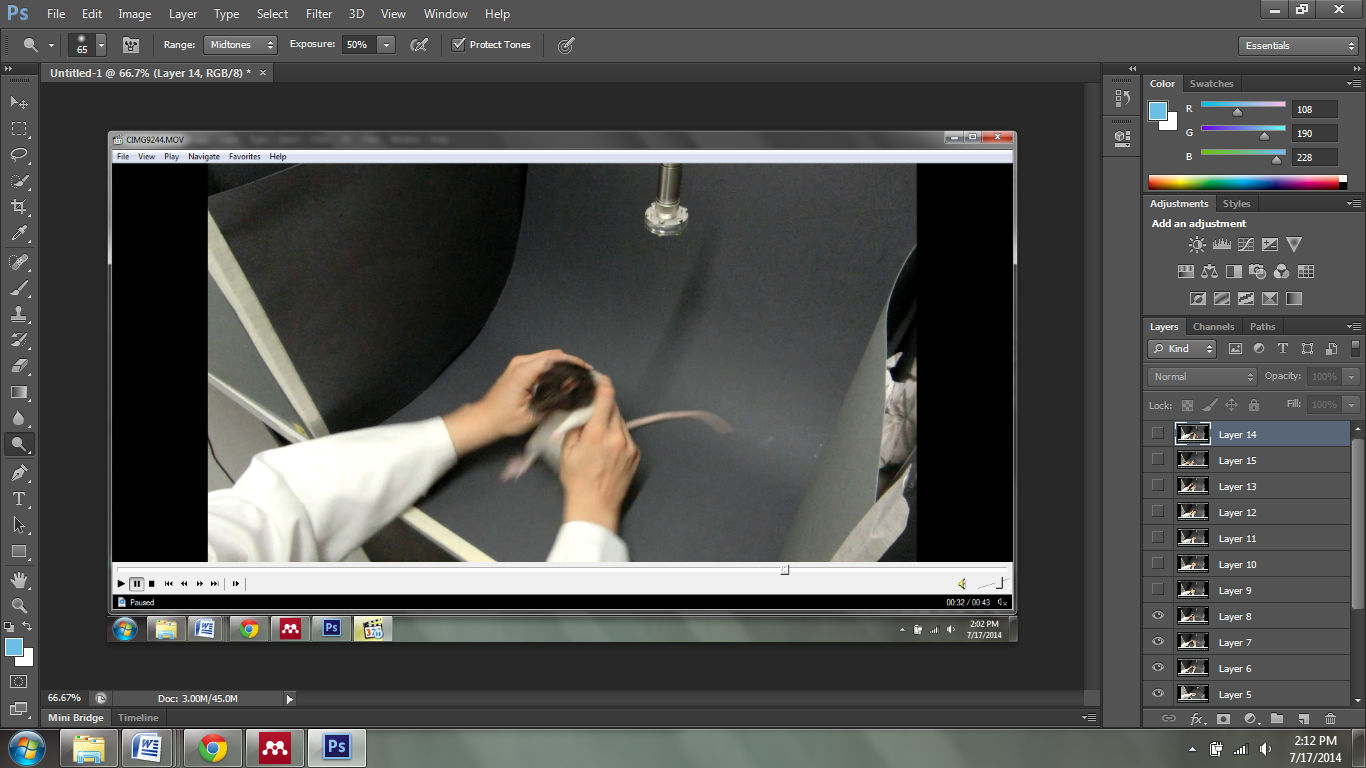

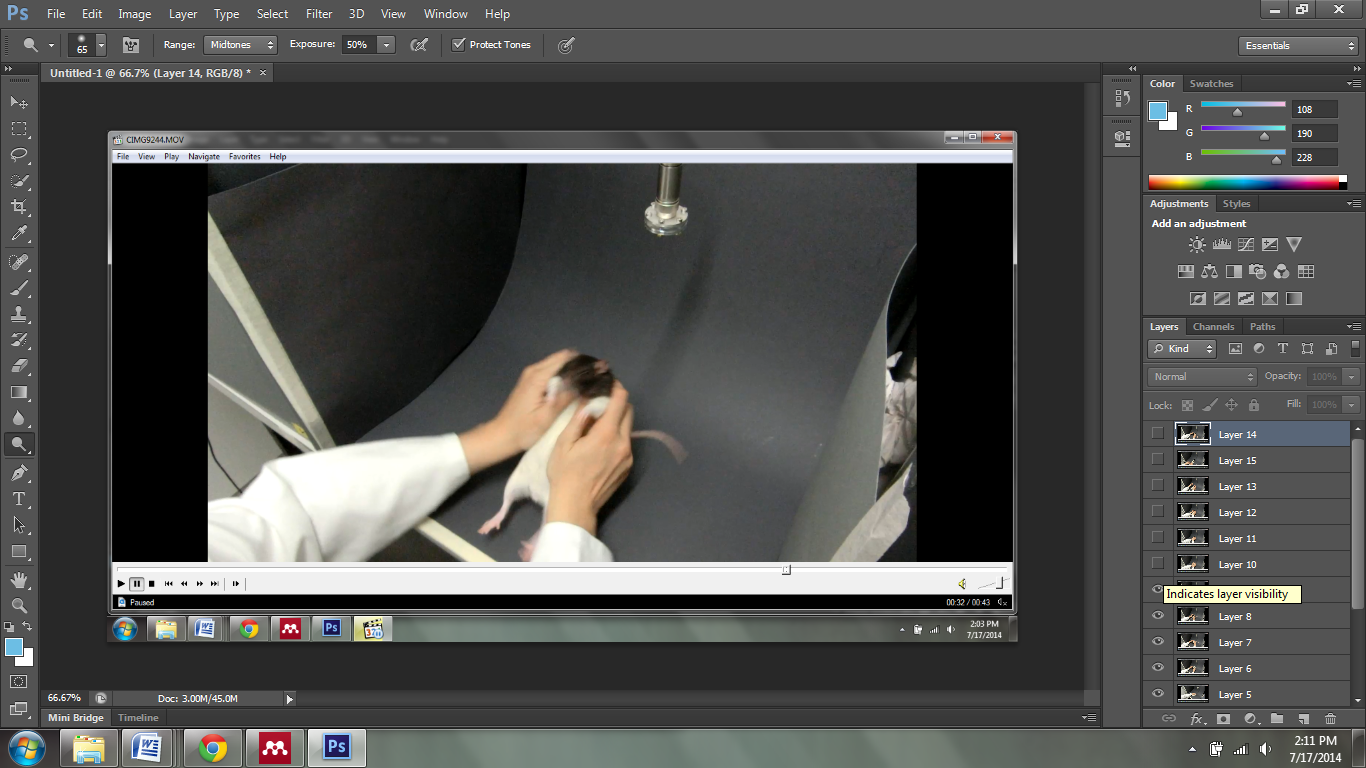


**Part 2:** Rat is tickled with the fingertips, by quick massaging and jiggling of the skin on the sides and nape of the neck. Listen for FM 50 kHz USVs.


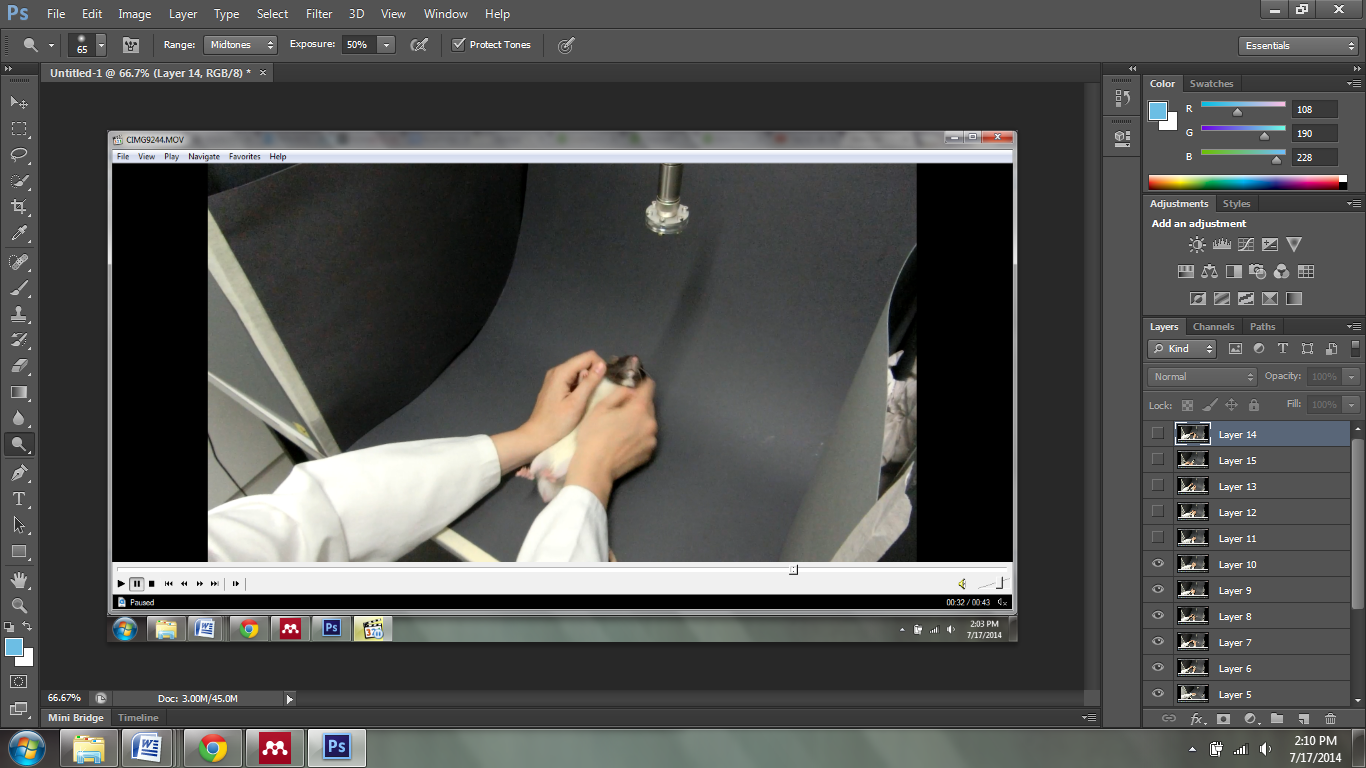

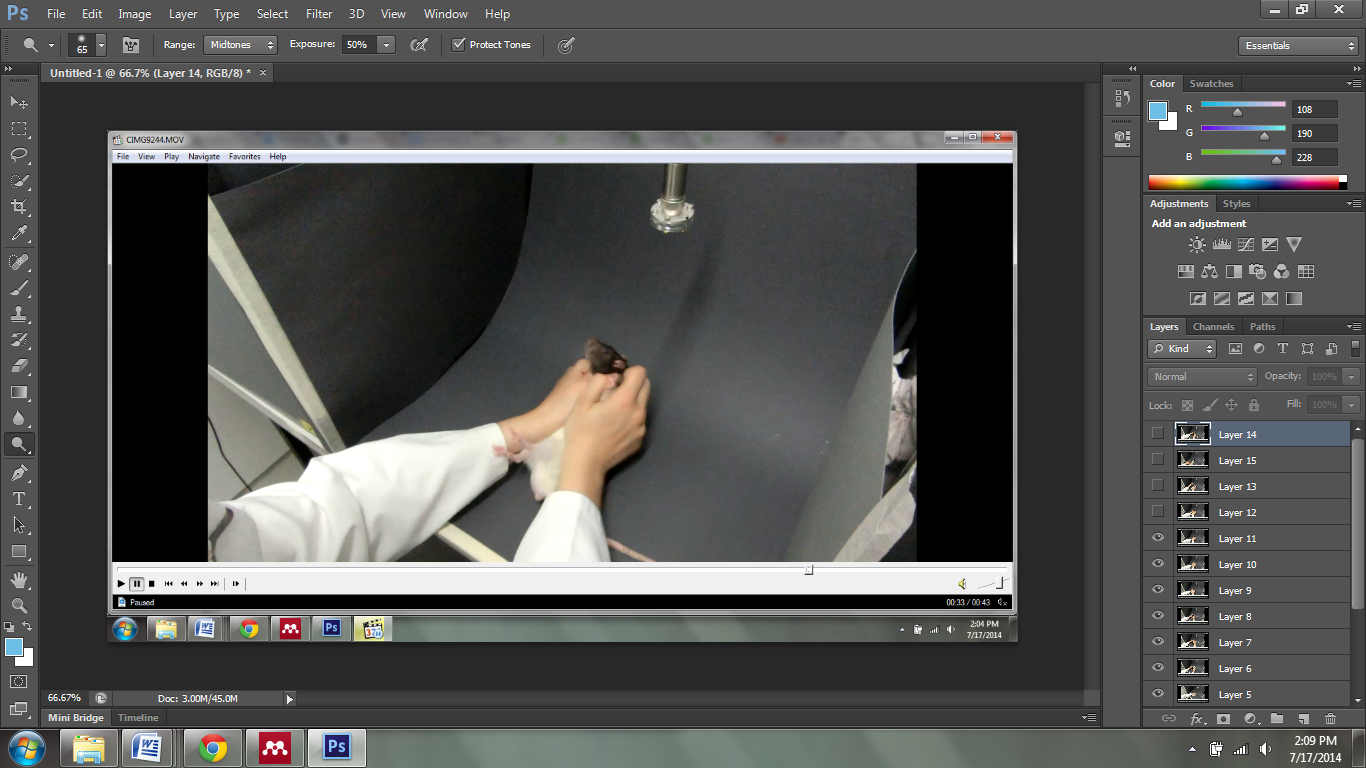


**Part 3:** Rat is released and it rights itself.


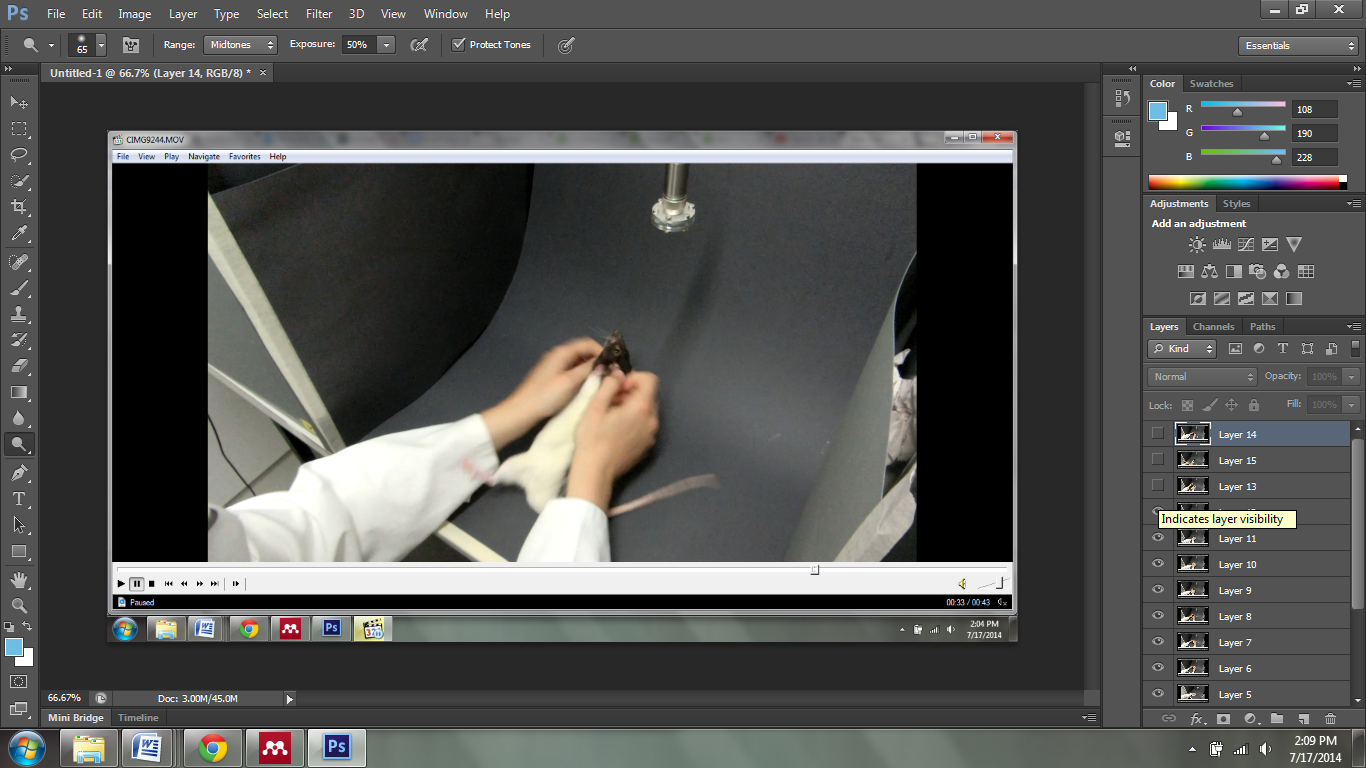

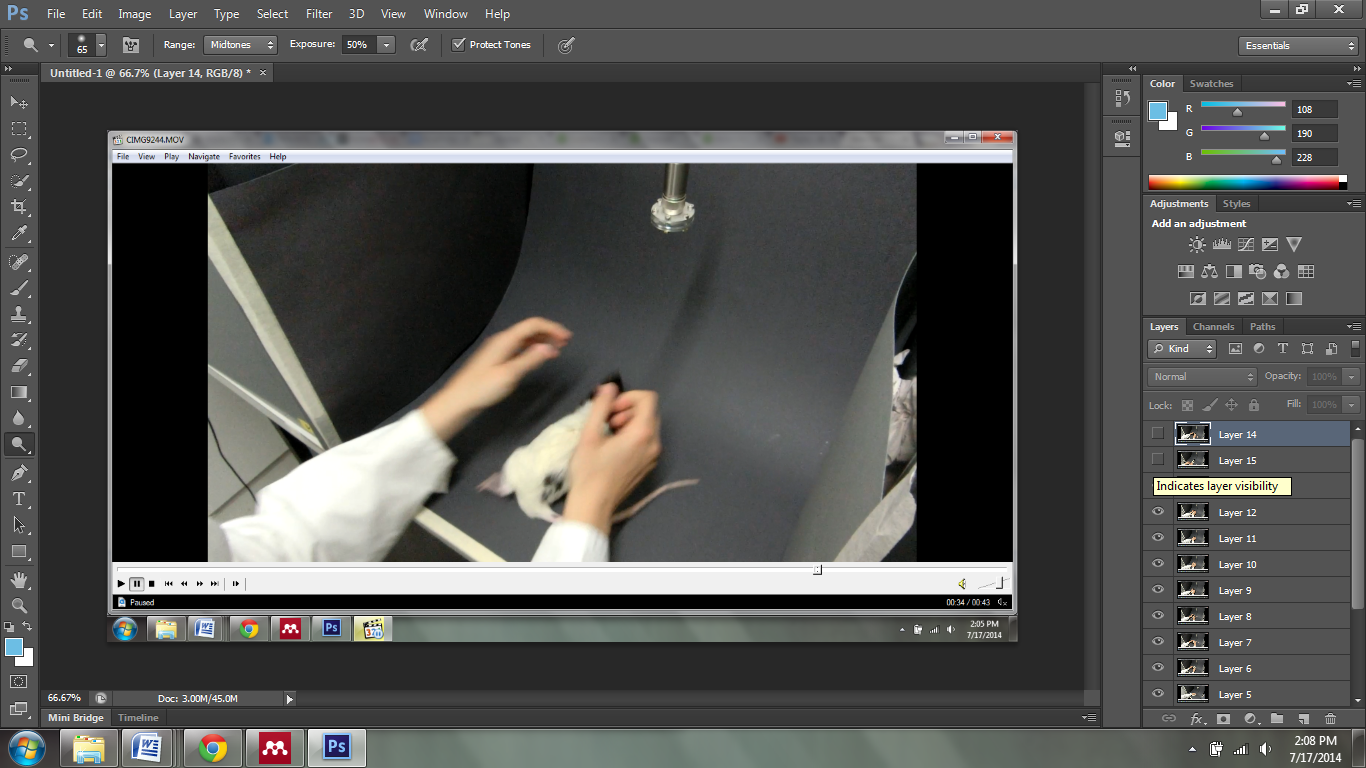

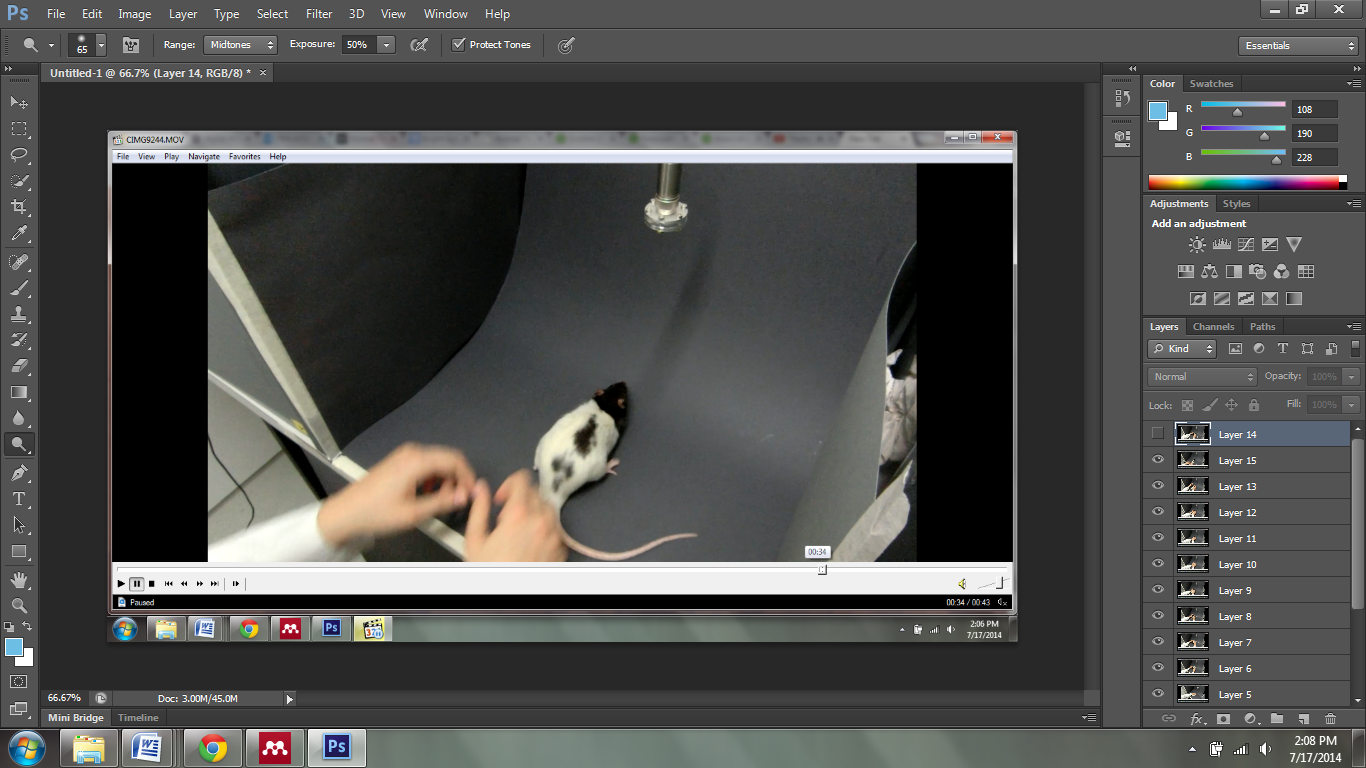

Supplement: S1 Appendix — (DOCX) [file pone.0166446.s001.docx]
