# Supplementary material for: Facial Indicators of Positive Emotions in Rats
Source: PLoS One. 2016 Nov 30;11(11):e0166446. doi: 10.1371/journal.pone.0166446 (PMC5130214; doi:10.1371/journal.pone.0166446)
Supplement: S2 Appendix — (DOCX) [file pone.0166446.s002.docx]

**S2 Appendix. Ear Colour Scoring Guide.**


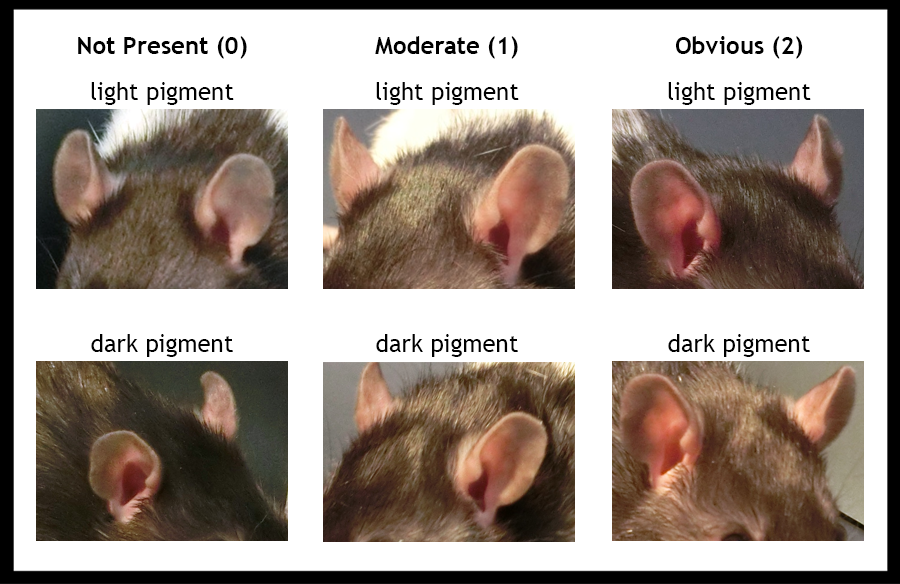


Ear colour should be determined by looking at the leading edge of the ear flap (dotted line) and lighter skin on the interior side of the flap (solid line). Both ears should be observed for colour, with preference given to the more visible ear. The ear canal, if visible, should not be used to determine colour, and shadows within folded over ears need to be considered as they may darken the skin inside the ear.
Lister Hooded rats have light grey pigmentation on the mid-to-outer edge of the ear which varies in shade between individuals. This pigment will change as the ear becomes pinker, but focus should be directed to determining the colour of the skin between the ear canal and pigmented edge, within the solid line.

**Score Rankings:**

**0 - Not Present**: ears are pale, appearing ashen or soft pink, with areas of pigmentation on the outer flap of the ear appearing light grey.

**1 - Moderate**: ears are slightly pink, intermediate between 0 and 2, with areas of pigmentation slightly darkened.

**2 - Obvious**: ears are flushed bright pink or dark rose, with areas of pigmentation appearing dark grey.
