# Supplementary material for: Facial Indicators of Positive Emotions in Rats
Source: PLoS One. 2016 Nov 30;11(11):e0166446. doi: 10.1371/journal.pone.0166446 (PMC5130214; doi:10.1371/journal.pone.0166446)
Supplement: S3 Appendix — (DOCX) [file pone.0166446.s003.docx]

**S3 Appendix. Visibility of the Nictitating Membrane Scoring Guide.**


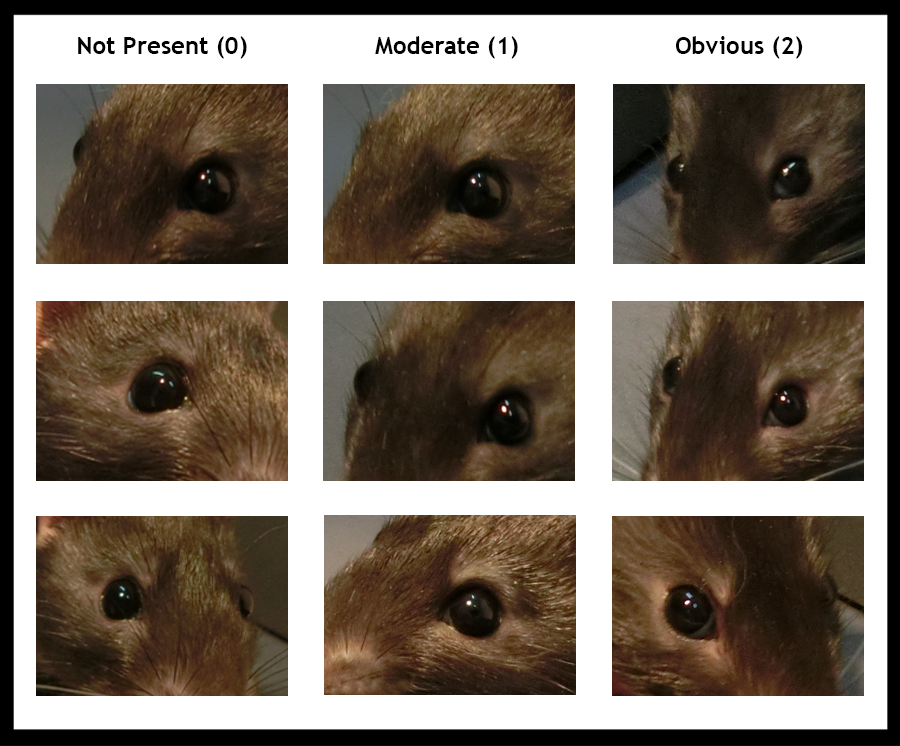


The nictitating membrane (third eyelid) is visible in the inner corner of the eye, separate from the tear duct. It is visible as a white crescent between the tear duct and iris.

**Score Rankings:**

**0 - Not Present**: no membrane visible

**1 - Moderate**: thin white line visible in the front of the eye

**2 - Obvious**: wide white line visible in the front of the eye
