# Supplementary material for: Facial Indicators of Positive Emotions in Rats
Source: PLoS One. 2016 Nov 30;11(11):e0166446. doi: 10.1371/journal.pone.0166446 (PMC5130214; doi:10.1371/journal.pone.0166446)

**S1 Figure.** **Test arena set-up.**
Test arena set-up in the Habituation/ Positive Treatment test room; the Contrast Treatment set-up was kept the same in a nearby room.


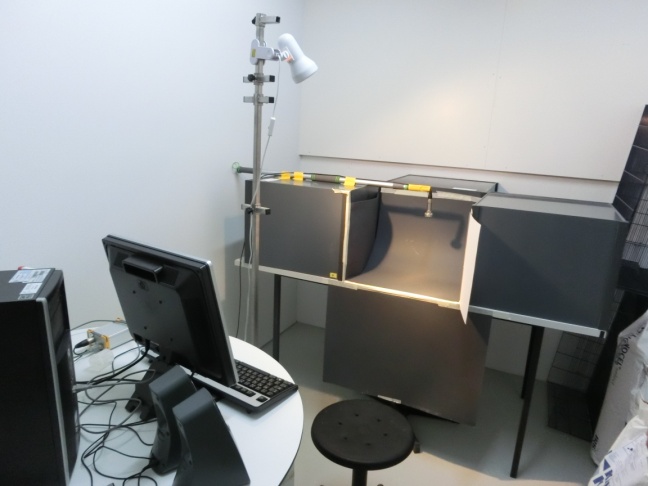

Supplement: S1 Fig — (DOCX) [file pone.0166446.s005.docx]
