# Supplementary material for: Facial Indicators of Positive Emotions in Rats
Source: PLoS One. 2016 Nov 30;11(11):e0166446. doi: 10.1371/journal.pone.0166446 (PMC5130214; doi:10.1371/journal.pone.0166446)
Supplement: S2 Fig — (DOCX) [file pone.0166446.s006.docx]

**S2 Figure. Examples of spectrograms of positive USVs emitted during one-handed (a) and two-handed (b) tickling.


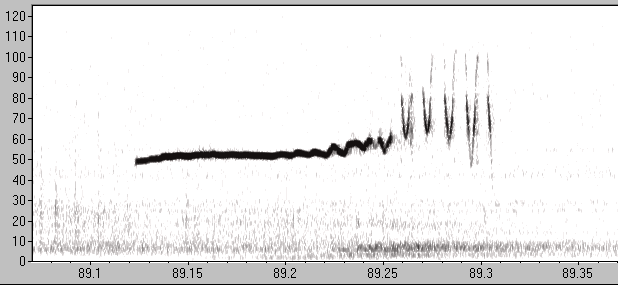
**

**a)** Spectrogram example of a positive frequency-modulated 50 kHz vocalisation emitted during one-handed tickling. The y axis indicates USV frequency (kHz) and the x axis indicates time (s).
Positive USVs were defined as vocalisations containing a trill component, regardless of whether they contained step, flat, ramp, or jump components [48]. Trills consisted of at least two "inverted-Us" of rapid frequency oscillations within a period of five milliseconds [48].


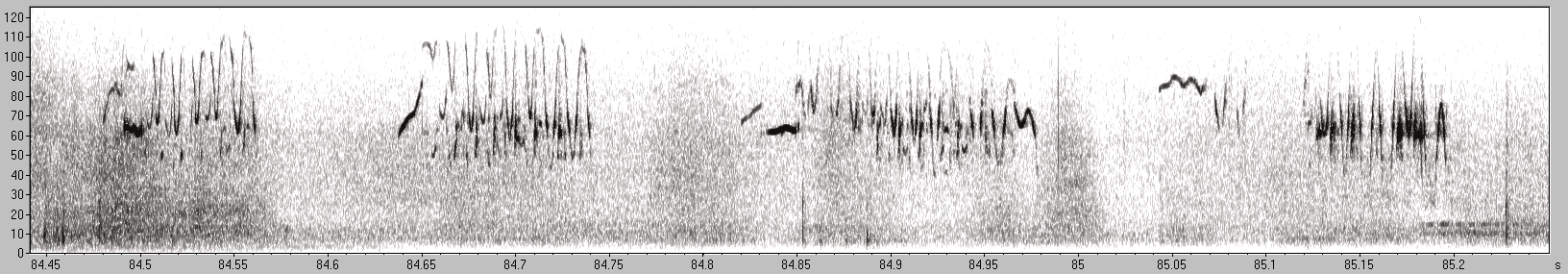


**b)** Spectrogram example of a positive frequency-modulated 50 kHz vocalisation emitted during two-handed tickling. The y axis indicates USV frequency (kHz) and the x axis indicates time (s).
From visual inspection of the spectrograms, the trill components emitted during two-handed tickling were as loud as or louder (i.e., more visible and darker trill line on the spectrogram) than those emitted during one-handed tickling.
